# Supplementary material for: Oral health in Brazil: What were the dental procedures performed in Primary Health Care?
Source: PLoS One. 2022 Jan 28;17(1):e0263257. doi: 10.1371/journal.pone.0263257 (PMC8797223; doi:10.1371/journal.pone.0263257)
Supplement: Attachment — Submitted filename: Response to reviewers.docx [file pone.0263257.s002.docx]

We would like to thank the reviewers for the excellent suggestions in order to improve our paper. The responses of each comment are listed below. All edition and revisions made are highlighted by red in the new file.

**Reviewer #1:**

The data were very old (2017-2018) and these data in present study is not contemporary, so these results don't reflect the current oral health requirements in Brazil.

*Response: The National Program for Improving Access and Quality of Primary Health Care (PMAQ-AB) is the largest health service evaluation program instituted in Brazil. The program ended in 2018 after three evaluation cycles. This study is based on national data, from the largest PHC evaluation program ever conducted in Brazil. These data refer to the last cycle of the PMAQ. Until this moment, there is no health service evaluation program under development in the country. So, nowadays, these are the data available that represents Brazilian national public program for oral health services, which reinforces the relevance of these results.*

Material method

Explain 5 macro region, mention which city in five region.

*Response: Thanks for the observations. We agree with the reviewer and the explanation about the Brazilian regions was included in Introduction section (7^th^ paragraph, 84 line; The differences between the regions are discussed from 230 Line in Discussion.*

**Reviewer #2:**

Thank you for submitting you manuscript. The manuscript presents good data about Brazilian national public program for oral health services. Public health professionals can use these valuable data in countries where they provide dental public health services.

Manuscript is organized and present sound interpretation of data, but needs some language editing and review to avoid confusion and make it more clear and readable. Especially for some terminologies that have been translated to English terms.

Here are some examples (in bold and underline):

69 three phases: accession and contractualisation, certification and recontratualisation, and a transversal strategic axis of development

*Response: Thanks for the observations. We agree with the reviewer. The text was rewritten and the change was made to “The PMAQ-AB is organized in three phases; adhesion and agreement, certification and re-agreement, and one strategic transverse axis of development...”*

78 OHTs performed individual preventive, restorative, and surgical procedures and emergencies; however

*Response: Thanks for the observations. We agree with the reviewer. The text was rewritten and the change was made to “OHTs performed emergency, preventive, restorative and surgical procedures; however…”*

132 performed by the OHTs, including preventive, restorative, surgical, and prosthetic 133 procedures and cancer monitoring.

*Response: Thanks for the observations. We agree with the reviewer. The text was rewritten and the change was made to “performed by the OHTs, including preventive, restorative/prosthetic, surgical and actions of cancer monitoring.”*

142 and cases in the community (80.5%). In addition, most OHTs did not realize biopsies

*Response: Thanks for the observations. We agree with the reviewer. The text was rewritten and the change was made to “**and cases in the population (80.5%).* *In addition, most OHTs did not perform biopsies”*

Also in Discussion:

The great accomplishment of basic oral health procedures by OHTs can be explained by the improvement in the infrastructure of dental offices, improvement in working conditions and higher qualification of professionals due to the increase in investments in oral health, provided by the National Oral Health Policy [16].

Comment: if you can elaborate more and exaplain what do you mean by the great accomplishment? Is it improvement in provided services compared to the 2nd cycle? Maybe mention some data from 2nd cycle to make it clear

*Response: Thanks for the observations and the opportunity to clarify this point. The better performance of OHTs, in basic procedures, is due to a greater investment in oral health, after the National Oral Health Policy. The text was rewritten: “The highest perform of basic oral health procedures by OHTs, like emergency, preventive, restorative and surgical procedures, can be explained by the improvement in the infrastructure of dental offices and working conditions, and higher qualification of professionals due to the increase in investments in oral health, provided by the National Oral Health Policy [17].”*

In this sense, the emergency dental care must be performed with a minimal use of high speed handpieces in order to avoid aerosols resulting from the use of manual instruments. Comment: you mean headpiece instrument?

*Response: Thanks for the observations and the opportunity to clarify this point. The text was rewritten:”* *In this sense, the emergency dental care must be performed with a minimal use of slow and high-speed handpieces in order to avoid aerosols, prioritizing the use of manual instruments.”*
