# Supplementary material for: Oral health in Brazil: What were the dental procedures performed in Primary Health Care?
Source: PLoS One. 2022 Jan 28;17(1):e0263257. doi: 10.1371/journal.pone.0263257 (PMC8797223; doi:10.1371/journal.pone.0263257)
Supplement: Attachment — Submitted filename: Response to reviewers_2.docx [file pone.0263257.s003.docx]

We would like to thank the reviewers for the excellent suggestions in order to improve our paper. The responses of each comment are listed below. All edition and revisions made are highlighted by red in the new file.

Additional Editor Comments

Please revise in accordance to reviewer's 3 comments before any further assessment of the manuscript.

6. Review Comments to the Author

Please use the space provided to explain your answers to the questions above. You may also include additional comments for the author, including concerns about dual publication, research ethics, or publication ethics. (Please upload your review as an attachment if it exceeds 20,000 characters)

Reviewer #2: (No Response)

Reviewer #3:This is a very interesting article. would suggest the authors to search if other similar articles were performed in other countries. In my opinion the materials and methods chapter has to be better explained. Please also in the discussion chapter try to find more recent published articles.

*Response: Thanks for the observations. There is no program for evaluation of quality of primary care similar to PMAQ, in other countries. However, we added three articles that discuss the systems of oral care in an international perspective (references 18, 19 and 20). The materials and methods chapter was reviewed to be more clear Recent published articles were added to. discussion chapter (references 18, 19, 20, 27 and 28).*
